# Clinical Learning Environment, Supervision, and Nurse Teacher Evaluation (CLES + T) Scale: Saudi Arabic Version—A Cross-Sectional Validation Study of Nursing Interns and Students

**DOI:** 10.1155/jonm/4483497

**Published:** 2025-11-07

**Authors:** Naif Hamdi Alanazi

**Affiliations:** Medical-Surgical Department, College of Nursing, King Saud University, Riyadh 12372, Saudi Arabia

**Keywords:** clinical experience, clinical learning environment, clinical practice, clinical supervision, nurse teacher evaluation, nursing intern, nursing student, Saudi Arabia

## Abstract

**Background:**

Robust instruments are essential to evaluate the effectiveness of the hospital setting as a clinical learning environment for nursing interns and students, which is a crucial part of nursing education.

**Purpose:**

This study aimed to present a Saudi Arabic version of Clinical Learning Environment, Supervision, and Nurse Teacher Evaluation (CLES + T-SA) scale and to explore the clinical learning environment, supervision, and nurse teacher evaluation of nursing interns and students during their clinical practice training.

**Methods:**

A convenience sample of 280 nursing interns and students was employed into this study. Exploratory factor analysis using principal axis factoring method and Varimax rotation was conducted to determine the structure of the scale's Saudi Arabic version.

**Results:**

The CLES + T-SA was successfully cross-culturally adapted and has a strong construct validity and reliability showing exceptional internal consistency (Cronbach's alpha = 0.96) with the following three subscales having Cronbach's alphas: clinical learning environment (0.94), supervision (0.94), and nurse teacher evaluation (0.96). Supervision is the most critical variable that influenced the clinical learning experience of nursing students.

**Conclusion:**

The CLES + T-SA version validly and reliably assessed CLES + T evaluation and can be used by nursing students in Arab Gulf countries and other Arabic-speaking nursing interns and students.

**Implications for Practice:**

Measures are needed to train and equip clinical supervisors in teaching, supervising, guiding, coaching, and facilitating the development of nursing students in providing compassionate, safe, and high-quality care during clinical practice. The findings of this study are significant to nursing academe and hospital administrators for them to formulate strategies that will enhance the clinical learning environment, supervision, and teaching of nursing students.

## 1. Introduction

Clinical practice is crucial and essential in nursing education [1–4]. Its aim is to support nursing students' transition from classroom instruction to real clinical practice [5, 6]. Due to the continuous changes in patients' healthcare needs and the increasingly complex healthcare environment [7], clinical practice facilitates the integration of nursing students' cognitive, psychomotor, and affective skills, thus playing a significant role in the development of these competencies [6, 8, 9]. To ensure that nursing students evolve into competent registered nurses, they must be educated and equipped with requisite competencies in delivering safe and high-quality care [10, 11]. This recommendation suggests that nursing education must focus on effectively training nursing students who can deliver good quality, safe, and compassionate care [3, 12], especially throughout the clinical practice [6, 8]. Thus, nursing clinical learning environments are particularly significant to develop nursing competence [13, 14] and to achieve good clinical practice of nursing students during their clinical training [6, 8, 15].

Meanwhile, the clinical learning of nursing students has been affected by lack of quality clinical sites, lack of qualified faculty, size of clinical groups (ratio of a faculty to students), restrictions in the number of students or limitations to students' experiences imposed by clinical agencies, and time-consuming nature of students learning multiple agency systems [16, 17]. In addition, socialization, communication, culture, relationships, and unmet expectations and aspirations are issues faced by nursing students in clinical learning practice [18, 19]. Similarly, findings from a systematic review exploring nursing students' appraisal of clinical practice placements, utilizing the Clinical Learning Environment, Supervision, and Nurse Teacher (CLES + T) scale, which included 21 studies, revealed challenges among nursing students during their clinical practice [8]. Nevertheless, the said review indicated that there is still room for improvement regarding students' satisfaction in clinical learning environments, as this is considered a crucial measure of program quality [8]. In Saudi Arabia, studies describing the state of the clinical learning environment among nursing students remain scant. Al-Anazi et al.'s [20] study was the first to evaluate clinical placements in Saudi Arabia using the CLES + T scale. Although the CLES + T was not translated to Arabic language, Saudi nursing students were generally satisfied with their clinical placements and found similar to students in other international studies regarding the appraisal of their clinical learning environments [20].

Globally, the Bachelor of Science in Nursing (BSN) program has a clinical nursing component wherein students are assigned to various clinical healthcare settings [13, 14]. In Saudi Arabia, nursing students with clinical practice included in their course are supervised by nurse teachers, known as nurse preceptors within Saudi Arabian context, from within the hospital placement [20]. The BSN curriculum in Saudi Arabia comprises theoretical and clinical components [14, 21]. After completing a 4-year basic program, nursing students undergo 1 year of nursing internship where they train intensively in various clinical nursing fields [22, 23]. From 2010 to 2012, the numbers of Saudi nursing graduates were between 423 and 461 with an average of 446 [7]. This figure had a significant increase, reaching 94,021 in the year 2023 [24]. Apparently, Saudi nursing graduates are still not sufficient to cope up with the growing needs of the kingdom resulting to reliance from expatriate nurses [25]. The clinical learning environment where Saudi nursing students (preinterns and interns) train must be explored to determine areas that must be improved and strengthened. This study sought to identify the state of the clinical learning environment and supervision on the basis of the experiences of Saudi nursing students with consideration of their cultural background during clinical and internship training. Therefore, this study aimed to culturally adapt and validate a Saudi Arabic version of CLES + T and explore the clinical learning environment, supervision, nurse teacher evaluation, and experiences of Saudi nursing students during clinical practice.

## 2. Methods

### 2.1. Study Design

A cross-sectional, correlational design was employed in this quantitative study. The study aimed to generate responses that were analyzed on the basis of numerical values using the Saudi Arabic version of the CLES + T evaluation scale.

### 2.2. Setting of the Study

The study was conducted in selected four tertiary training hospitals where preintern nursing students had their clinical training and intern nursing students were having their internship training. These training hospitals are equipped with adequate nursing units, which encompass medical and surgical areas for adult, maternity and pediatric populations, an emergency department featuring triage rooms, resuscitation rooms, and a fast-track unit, as well as critical care units, acute care units, isolation rooms, operating rooms, an outpatient department, and specialized dental department.

In the context of the current study, the term “clinical training” refers to the educational experience for nursing students who have not yet completed their studies at the College of Nursing and have undergone practical training in a hospital setting. For the supervision of preintern nursing students during clinical training, they are assigned to an instructor (nursing faculty) from the college and are supervised by a nurse preceptor (staff nurse) at the hospital. On average, the ratio of an instructor to preintern nursing students is 1:12, whereas the ratio of a nurse preceptor to preintern nursing students is 1:2 or in pairs. The total number of duty hours during clinical training when enrolled in a course with a clinical component is 6 hours, occurring once per week.

On the other hand, the term “internship training” signifies the experience of nursing students who have undergone a 1-year internship practice at a hospital facility. For the supervision of intern nursing students during internship training, they are assigned to a nurse educator from the nursing education department of the hospital while they are supervised by a nurse preceptor (staff nurse) within the nursing unit at the hospital. Typically, the average ratio of a nurse educator to intern nursing students stands at 1:8, while the ratio of a nurse preceptor to intern nursing students is 1:1. The total number of duty hours during internship training is 12 h, distributed to 4 days per week.

### 2.3. Participants and Sampling Design

A total of 300 nursing students (130 preinterns and 170 interns) were recruited as participants using convenience sampling method. The sample size was more than sufficient to have a medium effect size, at a power of 0.80 for *α* 0.05 to identify significant differences between two means and determine bivariate correlations on the basis of Cohen's [26] sample size determination model. Those who were officially enrolled and had their clinical or internship trainings in the first semester of academic year 2023-2024 were included. Nursing students who were non-Saudi, not enrolled, not having clinical training or internship practice, and those who participated in the pretest were excluded.

### 2.4. Research Instrument

The questionnaire used in this study has three parts. The first part comprised the profiles of the respondents that include: gender, age, clinical training level (preinternship or internship), occurrence of supervision, and frequency of separate private unscheduled supervision with a supervisor but without a nurse teacher.

The second part is the CLES + T evaluation scale [27, 28], which was translated into Arabic. The reliability of the original CLES + T subscales ranged between 0.77 and 0.96 on the basis of the principal component analysis. CLES + T is answerable by a Likert-type scale wherein participants rated their level of agreement to the items ranging from fully disagree (coded 1), disagree to some extent (coded 2), neither agree nor disagree (coded 3), agree to some extent (coded 4), or fully agree (coded 5). In this study, guidelines in interpreting, cross-cultural adaptation, and legalization of the CLES + T into Arabic were adapted [29]. The production of the Arabic version followed the following stages: translation, synthesis, back translation, review of experts, and pretest. The English version of the CLES + T scale was translated into Arabic by two independent experts, English to Arabic translators. The Arabic translations were validated by two independent expert translators from English to Arabic for consistency and readability. The translated Arabic version was back translated into English by two independent experts, Arabic to English translators. The back translations were found to be consistent with the original English version. The CLES + T Saudi Arabic version (CLES + T-SA) was pretested on 30 randomly selected nursing students from various year levels. The students were able to answer the CLES + T-SA version effortlessly between 15 and 20 min. The CLES + T-SA version is provided as supporting information for the current study.

### 2.5. Procedural and Ethical Considerations

Permission to translate into Arabic and use of the Saudi Arabic version of the CLES + T was obtained from the original copyright holder. Before the start of data collection, ethical approval from the Institutional Review Board (IRB) was obtained from King Saud University with Research Project Reference No.: 23/0689/IRB.

The preintern and intern nursing students were instructed to keep their identities completely anonymous while completing the questionnaires, and the researcher with the help from two assistant researchers took measures to safeguard their responses from being accessed by anyone outside the research team, keeping them confidential. To prevent any sharing of the collected data, the research team removed all identifying information, including demographic details, from the study results and disposed of this information 2 months after the current study concluded to ensure the confidentiality of the respondents.

In this study, participation was voluntary, and respondents were requested to sign an informed consent form that outlined the study's objectives and the terms and conditions of their involvement. They were explained that there was no monetary reward from participating in the study. They were also informed that they could withdraw from the study at any time they wished. Additionally, it was made clear that withdrawing from participation would not result in any physical harm or negative consequences at their training settings, and their registration at the College of Nursing at King Saud University would remain unaffected.

After obtaining the ethics approval, the structured questionnaire was administered to the respondents through paper-and-pencil/pen survey. To ensure that each respondent answered the survey only once, the responses to the questionnaire were systematically coded and recorded by the research team. Data were retrieved after the preintern and intern nursing students completed the survey. Data were gathered between October 2023 and November 2023.

### 2.6. Data Analysis

Data were processed and analyzed using IBM SPSS v.28 (Armonk, NY: IBM Corp). Data were screened for missing values, outliers, presence of multicollinearity, and singularity using regression procedures. Exploratory factor analysis using principal axis factoring method and Varimax rotation was conducted to determine the structure of the CLES + T-SA scale. The profiles of the participants were analyzed using descriptive statistics and presented using frequency distribution table. The two-tailed Mann–Whitney U test–Exact method was used to analyze the presence of significant differences in the perceived clinical learning environment, supervision, role of the nurse teacher, and clinical learning environment as a whole when grouped according to clinical training level. The Spearman rank correlation coefficient (*r*) was used to analyze the significant relationship between gender, age, training level, occurrence of supervision, frequency of separate private unscheduled supervision with a supervisor but without a nurse teacher and clinical learning environment, supervision, and nurse teacher overall clinical learning environment. The coefficient of determination (*r*^2^) was computed to determine the variables that explain variances in clinical learning environment, supervision, and nurse teacher overall clinical learning environment. Significant findings were inferred if *p* value was < 0.05.

## 3. Results

### 3.1. Exploratory Factor Analysis of CLES + T-SA

Data screening yielded 20 outliers which were not included in the final analysis (response rate = 93.3%, *N* = 280). The results of the data diagnostics did not show evidence of multicollinearity, singularity, or curvilinearity. The KMO measure of sampling adequacy yielded a result of 0.95, suggesting that a significant relationship exists between a dependent variable and single independent variable when the effects of other independent variables are held constant [30]. Bartlett's test of sphericity yielded *χ*^2^ 7967.34 and df 595, with *p* < 0.001 indicating the presence of correlations in the correlation matrix. The Scree test yielded three factors, which was the basis for the final number of factors for extraction. The three subscales that were generated were clinical learning environment with seventeen items, Eigenvalue of 15.83, 45.24% of the variance, factor loadings between 0.42 and 0.73, and Cronbach's *α* 0.94; nurse teacher evaluation with nine items, Eigenvalue of 3.46, 9.90% of the variance, factor loadings between 0.67 and 0.85, and Cronbach's *α* 0.96; supervision with nine items, Eigenvalue of 2.39, 6.84% of the variance, factor loadings between 0.61 and 0.79, and Cronbach's *α* 0.94; and the CLES + T evaluation scale-Saudi Arabic version as a whole with 35 items, Eigenvalue of 61.98, and Cronbach's *α* 0.96. The results of the factor analysis indicated that the construct validity and reliability based on interitem correlation coefficient (Cronbach's alpha) of the instrument are very good. The three subscales generated from the factor analysis were used to analyze the results of this study.

### 3.2. Profile of Nursing Students (Preinterns and Interns)

A total of 121 preinterns and 159 interns were included in this study (see Table 1). Most of the participants were female (87, 72% for preinterns; 104, 65% for interns), 20 to less than 25 years (94, 78% for preinterns; 132, 83% for interns). Nearly 25% (30/121) of the preinterns reported that the named supervisor changed during the placement, even though no change had been planned and the supervisor varied according to shift or place of work. Roughly 30% (48/159) of the interns reported that same supervisor had several students and was a group supervisor rather than an individual supervisor. Most (40, 33%) of the preinterns had separate private unscheduled supervision with a supervisor but without a nurse teacher once a week. Most (61, 38%) of the interns reported that they did not have separate private unscheduled supervision with a supervisor without a nurse teacher.

### 3.3. Perceptions of CLES + T and Significant Differences Between Preinterns and Interns

As shown in Table 2, the interns rated significantly higher (between *p* < 0.05 and *p* < 0.001) than preinterns on the following: staff learning to identify student by name, patients receiving individual nursing care, supervisor showing positive attitude toward supervision, supervision promoted learning, mutual interaction in supervisory relationship, supervisory relationship characterized by trust, and collective supervision. No significant difference exists (*p* < 0.05) between preinterns and interns in rating clinical learning environment, nurse teacher, and overall clinical learning environment.

### 3.4. Correlations Among Study Variables

As presented in Table 3, the occurrence of supervision and frequency of separate private unscheduled supervision with supervisor without nurse teacher is significantly associated with clinical learning environment (*p* < 0.01 and *p* < 0.05, respectively) and overall clinical learning environment (*p* < 0.05 and *p* < 0.01, respectively). Training level, occurrence of supervision, and frequency of separate private unscheduled supervision are significantly associated with supervision (*p* < 0.05, *p* < 0.001, and *p* < 0.01, respectively). Occurrence of supervision and frequency of separate private unscheduled supervision collectively explain 4% of the variance in clinical learning environment and 5% of the variance in overall clinical learning environment. Training level, occurrence of supervision, and frequency of separate private unscheduled supervision with a supervisor but without a nurse teacher collectively explain 10% of the variance in supervision.

## 4. Discussion

This study aimed to explore the perceptions of Saudi nursing interns and students in the clinical learning environment, supervision, and nurse teacher. To assess culturally sensitive perceptions of the clinical learning environment, supervision, and role of the nurse teacher during the clinical practice of nursing interns and students, the current study translated the CLES + T into Saudi Arabic language. The CLES + T-SA version was adapted culturally, revealing acceptable and sufficient factor loadings and reliability. In comparison, the psychometric evaluation of the CLES + T-SA version was found similar to other versions of CLES + T as a valid and reliable tool when used to evaluate the clinical learning environment of nursing students from other countries globally. These countries include Italy [31, 32], Cyprus [3], Spain [33], Nepal [34], Turkey [1], Slovenia and Croatia [15], Austria [35], and Korea [36]. Latest available psychometric evaluations of CLES + T show comparable results revealing good internal consistency of the scale with Cronbach's alpha of 0.94 in Turkey [37], Cronbach's alpha of 0.94 in Poland [38], and Cronbach's alpha of 0.94 in Hong Kong [39].

However, as the current study confirmed the validity of the CLES + T-SA for nursing students, as well as nursing interns who are newly graduated and still unlicensed nurses, the findings diverge from the Moroccan Arabic version of CLES + T [40], which identified five factors and was exclusively validated among nursing students. In addition, the Moroccan context of the CLES + T may not be applicable to the Saudi Arabian culture and the other five countries within the Arab Gulf region, also known as the Gulf Cooperation Council (GCC), which Saudi Arabia is a member nation.

The translation of the original version of the CLES + T into Saudi Arabic supports the need of nursing students to assess their clinical learning environments with considerations to their culture and native language, particularly in Arab Gulf countries like Saudi Arabia. This may pose great importance for Saudi nursing interns and students and their nurse teachers from the academe mainly because the nationalities of their preceptors in clinical placements are diverse. Diverse supervision situations among nursing students in clinical practice require cultural and linguistic support [41, 42]. This means that the role of the supervisor/mentor/preceptor is crucial in providing meaningful learning experiences to nursing students. This finding is supported by studies indicating that supportive supervision by staff and mentors toward students' learning contributed to the learning of nursing students [43, 44]. The results in this study are also supported by the findings of Chow et al. [45], indicating that as nursing students progress into higher year level—the internship phase in this study—they gain more experience, knowledge, skills, and also improvement in communicating with the staff and supervisor.

The results revealed that Saudi nursing interns and students perceived their experiences of the clinical learning environment more favorably as evidenced by their agreement especially in their relationship with the staff and supervisor, individualized nursing care to patients, positive attitude by the supervisor in supervising, and promotion of learning through supervision compared with preinterns. This positive rating may be attributed to the more intensive nature of the training and supervision that interns undergo. In addition, the actual length of clinical training for preinterns in this study sample was between 4 and 6 h in a week per course or 90 h per semester [20], which could be any of the following courses with clinical studies: adult health, pediatric, maternity, mental health, community, or critical care nursing. Meanwhile, nursing interns were required to undergo a 1-year comprehensive clinical training in 12-h shifts for 4 days a week. In comparison, nursing education is mainly a skill-based education in Nepal, as cited by Nepal et al. [34], and half of the curriculum (2460 clinical placement hours) in Austria, as cited by Mueller et al. [35]. Despite differing nursing programs, the satisfaction rates of clinical learning environment in this study sample are similar to those in previous studies in Europe [27, 28, 31–33, 35, 46–48], in Asia [34, 36], and in Arab Gulf countries [1, 20], and as reported in a recent systematic review that included 21 studies, predominantly conducted among nursing students in Sweden [8].

To better strengthen the case of the role of supervision in learning, it was found out that the occurrence of supervision and frequency of separate private unscheduled supervision with a supervisor but without a nurse teacher were important factors that significantly contributed to the clinical learning environment, supervision, and overall clinical learning environment. Interns were provided more frequent supervision than preinterns, as shown in this study. This difference explained why interns described positive learning experiences. Contrastingly, there were fewer occurrences of supervision to preinterns, which explained their negative learning experiences. The results showed that interns rated more positively than preinterns in most of the supervision-related variables. The results align with a previous study conducted in Slovakia, which indicated that nursing students engaged in individual supervision less frequently than the traditional group model of supervision [49]. Similar findings were documented in a prior study conducted in Sweden, which revealed that nursing students supervised under Model A (working in pairs) reported more positive clinical learning experiences than their counterparts in Model B, which followed to the individual supervision model [48].

### 4.1. Limitations of the Study

The limitations of the study included the issue of different study settings because nursing students (preinterns and interns) were undergoing clinical practice in four different hospitals and many dissimilar nursing units. This means that the findings may vary if analyzed at a hospital or nursing unit level rather than collectively. Additionally, if the study was conducted in a larger population and in a national level involving nursing students across the country, the results might be noteworthy. Further studies are needed to strengthen the usability of the CLES + T-SA scale. The study findings must be interpreted with caution, as they may not accurately reflect the perceptions of other nursing students undergoing clinical and internship training in privately owned healthcare facilities. Thus, these limitations restrict the generalizability of the findings of this study.

## 5. Conclusions

The CLES + T-SA scale, which was successfully cross-culturally adapted in this study, demonstrated strong construct validity and reliability. Hence, it can be used for future studies by Arabic-speaking nations in the GCC.

In this study, the findings showed that supervision is the most critical variable influencing the clinical learning experience of nursing students. Therefore, it is imperative to establish targeted measures designed to train and equip clinical supervisors and nurse preceptors with the essential knowledge, skills, and attitudes required for teaching, supervising, guiding, coaching, and facilitating the development of clinical and internship competencies of nursing students. Such measures will ensure that nursing students are competent in providing compassionate, safe, and high-quality care during their clinical practice.

## 6. Implications for Nursing Practice

The findings of this study are significant to nursing academe administrators to foster collaboration with their counterpart in the training hospitals and to enable them to formulate strategies that will enhance the clinical practice of nursing students (preinterns and interns). These findings will also help hospital nursing administrators, head nurses, clinical educators, and nurse preceptors of the training hospitals to reassess their clinical and internship training programs and to formulate strategies that will strengthen and promote positive clinical learning experience for nursing students. These strategies could be related to several aspects of clinical and internship training such as structured supervision (method/model), positive pedagogical atmosphere, peer-learning, and overall satisfaction of nursing students. Further exploration of the disparity in the supervision of preinterns and interns during their clinical training is warranted to formulate effective solutions in narrowing the gaps between theory and clinical practice of nursing students.

## Figures and Tables

**Table 1:** Profile of preinterns (nursing students) and nursing interns (*N* = 280).

	Preinterns (*n* = 121)		Interns (*n* = 159)	
	*f*	%	*f*	%
Gender				
Male	34	28.10	55	34.59
Female	87	71.90	104	65.41
Age				
Less than 20 years	17	14.05	2	1.25
20 to less than 25 years	94	77.69	132	83.02
25 to less than 30 years	9	7.43	21	13.21
30 to less than 35 years	1	0.83	4	2.52
Occurrence of supervision				
Did not have supervisor at all	4	3.31	7	4.40
A personal supervisor was named, but the relationship with this person did not work during the placement	28	23.14	18	11.32
The named supervisor changed during the placement, even though no change had been planned	30	24.79	15	9.43
The supervisor varied according to shift or place of work	30	24.79	47	29.56
Same supervisor had several students and was a group supervisor rather than an individual supervisor	12	9.92	48	30.19
A personal supervisor was named and our relationship worked during this placement	17	14.05	24	15.10
Frequency of separate private unscheduled supervision with the supervisor without nurse teacher				
Not at all	38	31.41	61	38.36
Once or twice only during the course	28	23.14	29	18.24
Once a week	40	33.06	29	18.24
Two times a week	10	8.26	23	14.47
Three or more times a week	5	4.13	17	10.69

**Table 2:** Perceptions of clinical learning environment, supervision, and nurse teacher evaluation and significant differences between preinterns and interns (*N* = 280).

	Mean rank		Mann–Whitney *U* test	
	Preintern (*n* = 121)	Intern (*n* = 159)	*Z*	*p* value
Occurrence of supervision	120.28	155.89	−3.725	< 0.001^∗∗∗^
The staff learned to know the student by their personal names	127.25	150.58	−2.479	0.013^∗^
Patients received individual nursing care	122.49	154.21	−3.390	0.001^∗∗^
My supervisor showed a positive attitude toward supervision	121.77	154.75	−3.535	< 0.001^∗∗∗^
The supervision promoted my learning	129.69	148.73	−2.029	0.042^∗^
There was a mutual interaction in the supervisory relationship	129.17	149.12	−2.126	0.034^∗^
The supervisory relationship was characterized by a sense of trust	128.27	149.81	−2.295	0.022^∗^
Clinical learning environment	139.14	141.54	−0.264	0.792
Supervision	128.53	149.61	−2.161	0.031^∗^
Nurse teacher	142.21	139.20	−0.309	0.757
Overall clinical learning environment	141.99	139.36	−290	0.772

^∗^Significant at *p* < 0.05.

^∗∗^Significant at *p* < 0.01.

^∗∗∗^Significant at *p* < 0.001.

**Table 3:** Correlations among study variables (*N* = 280).

	Clinical learning environment		Supervision		Nurse teacher		Overall clinical learning environment	
	*r*	*r* ^2^	*r*	*r* ^2^	*r*	*r* ^2^	*r*	*r* ^2^
Gender	−0.011		−0.060		0.027		0.017	
Age	−0.054		−0.017		−0.075		−0.101	
Training level	0.016		0.129^∗^	0.02	−0.018		−0.017	
Occurrence of supervision	0.166^∗∗^	0.03	0.215^∗∗∗^	0.05	0.090		0.139^∗^	0.02
Frequency of separate private unscheduled supervision with supervisor without nurse teacher	0.117^∗^	0.01	0.179^∗∗^	0.03	0.107		0.168^∗∗^	0.03

^∗^Significant at *p* < 0.05.

^∗∗^Significant at *p* < 0.01.

^∗∗∗^Significant at *p* < 0.001.

## Data Availability

The data that support the findings of this study are available on request from the corresponding author. The data are not publicly available due to privacy or ethical restrictions.

## Supporting Information

Clinical Learning Environment, Supervision, and Nurse Teacher Evaluation Scale–Saudi Arabic Version (CLES + T-SA).
