# Supplementary material for: Clinical Learning Environment, Supervision, and Nurse Teacher Evaluation (CLES + T) Scale: Saudi Arabic Version—A Cross-Sectional Validation Study of Nursing Interns and Students
Source: J Nurs Manag. 2025 Nov 7;2025:4483497. doi: 10.1155/jonm/4483497 (PMC12618126; doi:10.1155/jonm/4483497)
Supplement: Supporting Information — Additional supporting information can be found online in the Supporting Information section. [file 4483497.f1.doc]

| **(بيئة التعلم السريرية, والإشراف ومدرس التمريض)**  **(بيئة التعلم السريرية والإشراف + مدرس) مقياس التقييم (الإصدار العربي)**  **CLINICAL LEARNING ENVIRONMENT, SUPERVISION AND NURSE TEACHER**  **(CLES+T) Evaluation Scale (Saudi Arabic Version)** |
| --- |

|  | |  |
| --- | --- | --- |
| **The Learning Environment**:  The following are statements concerning the learning environment, supervision and the role of nurse teacher. The statements are grouped according to their categories.  For each statement, please put a check mark ( **/** ) on the number that best describes your own opinion based on the following evaluation scale: | **بيئة التعلم**  العبارات التالية عبارات تتعلق ببيئة التعلم والإشراف ودور مدرس التمريض. وقد تم تجميع العبارات وفق فئتها  بالنسبة لكل عبارة، الرجاء وضع علامة التحديد  على الرقم الذي يعبر عن رأيك بناءً على مقياس التقييم التالي: ( **/)** | |

| *مقياس التقييم:* | Evaluation scale*:* |
| --- | --- |
| 1 = لا أوافق تماماً | 1 = fully disagree |
| 2 = لا أوافق إلى حدٍ ما | 2 = disagree to some extent |
| 3 = لا أوافق ولا أرفض | 3 = neither agree nor disagree |
| 4 = أوافق إلى حدٍ ما | 4 = agree to some extent |
| 5 = أوافق تماماً | 5 = fully agree |

| **Learning atmosphere** | | | | **مجال التعلم** | | | | | | | | | | | | | | | | |
| --- | --- | --- | --- | --- | --- | --- | --- | --- | --- | --- | --- | --- | --- | --- | --- | --- | --- | --- | --- | --- |
| The staff were easy to approach  (كان من السهل الوصول إلى فريق العمل) | | | | 1 | | | | 2 | | | 3 | | | | 4 | | | | 5 | |
| I felt comfortable going to the ward at the start of my shift  (شعرت بارتياح عندما ذهبت إلى الجناح مع بدء مناوبتي) | | | | 1 | | | | 2 | | | 3 | | | | 4 | | | | 5 | |
| During staff meetings (for example, before shifts) I felt comfortable taking part in the discussions  (خلال اجتماعات الموظفين (على سبيل المثال، قبل المناوبة) شعرت بارتياح لمشاركتي في المناقشات) | | | | 1 | | | | 2 | | | 3 | | | | 4 | | | | 5 | |
| There was a positive atmosphere on the ward  (كانت البيئة إيجابية بالجناح) | | | | 1 | | | | 2 | | | 3 | | | | 4 | | | | 5 | |
| The staff were generally interested in student supervision  (كان الموظفين مهتمين بصورة عامة بالإشراف على الطلاب) | | | | 1 | | | | 2 | | | 3 | | | | 4 | | | | 5 | |
| The staff learned to know the students by their personal names  (تعلم الموظفين طريقة التعرف على الطلاب من خلال أسماؤهم الشخصية) | | | | 1 | | | | 2 | | | 3 | | | | 4 | | | | 5 | |
| There were sufficient meaningful learning situations on the ward  (كانت هناك مواقف تعليمية مفيدة في الجناح) | | | | 1 | | | | 2 | | | 3 | | | | 4 | | | | 5 | |
| The learning situations were multi-dimensional in terms of content  (كانت المواقف التعليمية متعددة الأبعاد من حيث المحتوى) | | | | 1 | | | | 2 | | | 3 | | | | 4 | | | | 5 | |
| The ward can be regarded as a good learning environment  (يمكن اعتبار الجناح بيئة تعليمية جيدة) | | | | 1 | | | | 2 | | | 3 | | | | 4 | | | | 5 | |
| **Leadership style of the ward manager (WM)**: | | | **نمط القيادة لمدير الجناح** | | | | | | | | | | | | | | | | | |
| The WM regarded the staff on her/his ward as a key resource  (مدير الجناح ينظر إلى الموظفين العاملين في الجناح التابع له/ لها على أنهم أحد الموارد الأساسية) | | | 1 | | | | 2 | | | 3 | | | 4 | | | | 5 | | | |
| The WM was a team member  (كان مدير الجناح أحد أعضاء الفريق) | | | 1 | | | | 2 | | | 3 | | | 4 | | | | 5 | | | |
| Feedback from the WM could easily be considered as a learning situation  (أي تعليق يصدر من جانب مدير الجناح يمكن اعتباره بسهولة موقف تعليمي) | | | 1 | | | | 2 | | | 3 | | | 4 | | | | 5 | | | |
| The efforts of individual employees were appreciated  (تم تقدير الجهود التي بذلها الموظفين) | | | 1 | | | | 2 | | | 3 | | | 4 | | | | 5 | | | |
|  | | |  | | | |  | | |  | | |  | | | |  | | | |
| **Nursing care at the ward**: (**الرعاية التمريضية في الجناح**) | | | | | | | | | | | | | | | | | | | | |
| The ward’s nursing philosophy was clearly defined  (تم إيضاح فلسفة التمريض بالأجنحة بصورة واضحة) | | | 1 | | | | 2 | | | 3 | | | 4 | | | | 5 | | | |
| Patients received individual nursing care  (تلقى المرضى رعاية تمريضية فردية) | | | 1 | | | | 2 | | | 3 | | | 4 | | | | 5 | | | |
| There were no problems in the information flow related to patients’ care  (لم تكن هناك أية مشاكل في نقل المعلومات المتعلقة برعاية المرضى) | | | 1 | | | | 2 | | | 3 | | | 4 | | | | 5 | | | |
| Documentation of nursing (for example: nursing plans, daily recording of nursing procedures etc.) was clear  (كانت وثائق التمريض (على سبيل المثال، خطط التمريض، السجلات اليومية لإجراءات التمريض، إلخ... واضحة) | | | 1 | | | | 2 | | | 3 | | | 4 | | | | 5 | | | |
|  | | | | | | | | | | | | | | | | | | | | |
| **The supervisory relationship:** (**العلاقة الإشرافية**) | | | | | | | | | | | | | | | | | | | | |
| في هذا القسم، يشير مفهوم الإشراف إلى توجيه ودعم وتقييم طلاب التمريض من قبل طاقم التمريض السريري. يمكن أن تتم عملية الإشراف على شكل إشراف فردي أو إشراف جماعي (أو في فريق).  In this section, the concept of **supervision** refers to guiding, supporting and assessing of student nurses made by clinical staff nurses. **Supervision** can occur as **individual supervision, or as group (or team) supervision**  مفهوم المعلم الخاص أو المدرب يعني أي مشرف شخصي معين  The concept of **mentor or preceptor** means a designated personal supervisor.   | What is the job title of your clinical supervisor: | ما مسمى وظيفة مشرفك السريري: | | --- | --- | | 1 Nurse | 1. ممرض | | 2 Nurse Specialist | 2. أخصائي تمريض | | 3 Nurse Educator | 3. معلم تمريض | | 4 Assistant ward manager | 4. مساعد مدير جناح | | 5 Ward Manager | 5. مدير جناح | | 6 Other (please specify) | 6. أسماء أخرى (الرجاء التحديد) | | Occurrence of supervision: (Check one only) | تنفيذ الإشراف: (حدد اختيار واحد فقط | | 1 I did not have a supervisor at all | 1. ليس لدي مشرف على الإطلاق | | 2 A personal supervisor was named, but the relationship with this person  did not work during the placement | 2. تم تعيين مشرف شخصي، لكن العلاقة مع هذا الشخص لم تسر على ما يرام خلال التدريب | | 3 The named supervisor changed during the placement, even though  no change had been planned | 3 تغير المشرف المعين خلال التدريب، على الرغم من أنه لم يكن مخططًا إجراء أي تغيير | | 4 The supervisor varied according to shift or place of work | 4. تغير المشرف تبعًا للمناوبة أو مكان العمل | | 5 Same supervisor had several students and was a group supervisor rather  than an individual supervisor | 5. يتولى نفس المشرف أمر الإشراف على العديد من الطلاب وقد كان مشرف مجموعة أكثر من كونه مشرفًا فرديًا | | 6 A personal supervisor was named and our relationship worked  during this placement | 6. تم تعيين مشرف شخصي وقد نجحت العلاقة بيننا خلال التدريب | | 7 Other method of supervision, please specify? | 7. طرق أخرى للإشراف، الرجاء التحديد؟ |  | How often did you have separate private unscheduled supervision with the supervisor without nurse teacher: | كم مرة في الغالب حصلت على إشراف منفصل خاص وغير مخطط له مع المشرف بدون مدرس تمريض: | | --- | --- | | 1 Not at all | 1. لم أحظى بذلك على الإطلاق | | 2 Once or twice only during the course | 2. مرة أو مرتين فقط خلال التدريب | | 3 Once a week | 3. مرة في الأسبوع | | 4 Two times a week | 4. مرتان في الأسبوع | | 5 Three or more times a week | 5. ثلاث مرات في الأسبوع أو أكثر |   **The Supervisory Relationship** (**العلاقة الإشرافية**)   | تمثل العبارات التالية عبارات تتعلق بالعلاقة الإشرافية  The following are statements concerning the supervisory relationship.  بالنسبة لكل عبارة، الرجاء وضع علامة التحديد ( **/** ) على الرقم الذي يعبر عن رأيك بناءً على مقياس التقييم التالي)  For each statement, please put a check mark ( **/** ) on the number that best describes your own opinion based on the following evaluation scale: | | --- |  | مقياس التقييم: | Evaluation scale: | | --- | --- | | 1 = لا أوافق تماماً | 1 = fully disagree | | 2 = لا أوافق إلى حدٍ ما | 2 = disagree to some extent | | 3 = لا أوافق ولا أرفض | 3 = neither agree nor disagree | | 4 = أوافق إلى حدٍ ما | 4 = agree to some extent | | 5 = أوافق تماماً | 5 = fully agree | | | | | | | | | | | | | | | | | | | | | |
|  | | | | | | | | | | | | | | | | | | | | |
| My supervisor showed a positive attitude towards supervision  (أبدى مشرفي توجهًا إيجابيًا تجاه عملية الإشراف) | | | | | | 1 | | | 2 | | | 3 | | | | 4 | | | | 5 |
| I felt that I received individual supervision  (شعرت بأنني حصلت على إشراف فردي) | | | | | | 1 | | | 2 | | | 3 | | | | 4 | | | | 5 |
| I continuously received feedback from my supervisor  (تلقيت ردود الفعل بصورة مستمرة من مشرفي) | | | | | | 1 | | | 2 | | | 3 | | | | 4 | | | | 5 |
| Overall, I am satisfied with the supervision I received  (بصورة عامة، أنا راضٍ عن الإشراف الذي حصلت عليه) | | | | | | 1 | | | 2 | | | 3 | | | | 4 | | | | 5 |
| The supervision was based on a relationship of equality  (كانت عملية الإشراف قائمة على علاقة المساواة) | | | | | | 1 | | | 2 | | | 3 | | | | 4 | | | | 5 |
| The supervision promoted my learning  (ارتقت عملية الإشراف بمستوى تعليمي) | | | | | | 1 | | | 2 | | | 3 | | | | 4 | | | | 5 |
| There was a mutual interaction in the supervisory relationship  (كان هناك تفاعل متبادل في العلاقة الإشرافية) | | | | | | 1 | | | 2 | | | 3 | | | | 4 | | | | 5 |
| Mutual respect and approval prevailed in the supervisory relationship  (الاحترام والتوافق المتبادل ساد في العلاقة الإشرافية) | | | | | | 1 | | | 2 | | | 3 | | | | 4 | | | | 5 |
| The supervisory relationship was characterized by a sense of trust  (تميزت العلاقة الإشرافية بالثقة) | | | | | | 1 | | | 2 | | | 3 | | | | 4 | | | | 5 |
|  | | | | | | | | | | | | | | | | | | | | |
| | دور مدرس التمريض | Role of the Nurse Teacher | | --- | --- | | (مدرس التمريض هو محاضر يتم تعيينه من جانب الجامعة أو الكلية متعددة التقنيات والذي يكون مسؤول عن تدريس النظري والسريري)  (تمثل العبارات التالية عبارات تتعلق بمدرس التمريض. وقد تم تجميع العبارات وفق فئتها)  بالنسبة لكل عبارة، الرجاء وضع علامة التحديد ( / ) على الرقم الذي يعبر عن رأيك بناءً على مقياس التقييم التالي) | **Nurse teacher** is a lecturer employed by the University or Polytechnic who is responsible to both theoretical and clinical teaching.  The following are statements concerning the nurse teacher. The statements are grouped according to their categories.  For each statement, please put a check mark ( / ) on the number that best describes your own opinion based on the following evaluation scale: |  |  |  | | --- | --- | | Evaluation scale: | مقياس التقييم: | | 1 = fully disagree | 1 = لا أوافق تماماً | | 2 = disagree to some extent | 2 = لا أوافق إلى حدٍ ما | | 3 = neither agree nor disagree | 3 = لا أوافق ولا أرفض | | 4 = agree to some extent | 4 = أوافق إلى حدٍ ما | | 5 = fully agree | 5 = أوافق تماماً |  | مدرس التمريض من حيث تحقيق التكامل بين النظري والعملي | **Nurse teacher as enabling the integration of theory and practice**: | | --- | --- | | | | | | | | | | | | | | | | | | | | | |
| In my opinion, the nurse teacher was capable to integrate theoretical knowledge and everyday practice of nursing  (في رأيي، تمكن مدرس التمريض من تحقيق التكامل بين المعرفة النظرية والممارسة اليومية لمهنة التمريض) | | | 1 | | | | 2 | | | | 3 | | | 4 | | | | 5 | | |
| The teacher was capable of operationalizing the learning goals of this clinical placement  (تمكن مدرس التمريض من تنشيط أهداف التعليم المنشود من هذا التدريب السريري) | | | 1 | | | | 2 | | | | 3 | | | 4 | | | | 5 | | |
| The nurse teacher helped me to reduce the theory-practice gap  (قدم مدرس التمريض المساعدة لي في تقليل الفجوة الكائنة بين النظري والعملي) | | | 1 | | | | 2 | | | | 3 | | | 4 | | | | 5 | | |
|  | | | | | | | | | | | | | | | | | | | | |
|  | | | | | | | | | | | | | | | | | | | | |
| **Cooperation between placement staff and nurse teacher**: | | **التعاون بين الموظفين بالتدريب ومدرس التمريض** | | | | | | | | | | | | | | | | | | |
| The nurse teacher was like a member of the nursing team  (كان مدرس التمريض كعضو في فريق التمريض) | | 1 | | | 2 | | | | 3 | | | 4 | | | | 5 | | | | |
| The nurse teacher was capable to give his or her teaching expertise to the clinical team  (تمكن مدرس التمريض من منح الفريق السريري ما يتمتع به من خبرة في التدريس) | | 1 | | | 2 | | | | 3 | | | 4 | | | | 5 | | | | |
| The nurse teacher and the clinical team worked together in supporting my learning  (عمل مدرس التمريض والفريق السريري معًا على دعم تعليمي) | | 1 | | | 2 | | | | 3 | | | 4 | | | | 5 | | | | |
|  | | | | | | | | | | | | | | | | | | | | |
| **Relationship among student, mentor and nurse teacher**: | **(العلاقة فيما بين الطالب والمعلم الخاص ومدرس التمريض**) | | | | | | | | | | | | | | | | | | | |
| The common meetings between myself, mentor and nurse teacher were comfortable experience  (كانت الاجتماعات المشتركة التي جمعت بيني وبين المعلم الخاص ومدرس التمريض تجربة مريحة) | 1 | | | | 2 | | | | 3 | | | 4 | | | | 5 | | | | |
| In our common meetings I felt that we are colleagues  (في اجتماعاتنا المشتركة، شعرت بأننا زملاء عمل) | 1 | | | | 2 | | | | 3 | | | 4 | | | | 5 | | | | |
| Focus on the meetings was in my learning needs  (مثل التركيز على الاجتماعات عنصرًا ضمن احتياجات التعلم الخاصة بي) | 1 | | | | 2 | | | | 3 | | | 4 | | | | 5 | | | | |

|  |  |
| --- | --- |
